# Optimal Base Station Density of Dense Network: From the Viewpoint of Interference and Load

**DOI:** 10.3390/s17092077

**Published:** 2017-09-11

**Authors:** Jianyuan Feng, Zhiyong Feng

**Affiliations:** School of Information and Communication Engineering, Beijing University of Posts and Telecommunications (BUPT), Beijing 100876, China; fjy919@bupt.edu.cn

**Keywords:** network densification, heterogeneous network, interference, load, base station density

## Abstract

Network densification is attracting increasing attention recently due to its ability to improve network capacity by spatial reuse and relieve congestion by offloading. However, excessive densification and aggressive offloading can also cause the degradation of network performance due to problems of interference and load. In this paper, with consideration of load issues, we study the optimal base station density that maximizes the throughput of the network. The expected link rate and the utilization ratio of the contention-based channel are derived as the functions of base station density using the Poisson Point Process (PPP) and Markov Chain. They reveal the rules of deployment. Based on these results, we obtain the throughput of the network and indicate the optimal deployment density under different network conditions. Extensive simulations are conducted to validate our analysis and show the substantial performance gain obtained by the proposed deployment scheme. These results can provide guidance for the network densification.

## 1. Introduction

We are embracing a booming era of information technology, where all people and industries are powered to reach their full potential. In the coming few years, smart devices of an increasingly powerful scope will connect to wireless networks, allowing the opportunity to use extremely diverse multimedia services. The technical reports from Ericsson and Qualcomm [1,2] predicted that the number of devices connected to wireless networks would soar to 28 billion by 2021 and the mobile data traffic in 2020 will be 1000 times greater than that of the 2010 baseline. These trends show a high traffic density in the near future. To triumph over this formidable challenge, International Telecommunication Union-Radiocommunication Sector (ITU-R) has defined Enhance Mobile Broadband (eMBB) as one of three typical scenarios of 5G (5th generation wireless systems) [3]. According to the report from IMT-2020 promotion group of China, the 5G network is expected to provide tens of Tbps/km${}^{2}$ traffic volume density [4].

As a key solution to high traffic density, network densification is proposed to deploy ultra-dense heterogeneous networks (HetNets) [5]. Network densification has many advantages. It improves the area spectral efficiency by reusing the spectrum extensively over geographic areas, which effectively deals with the spectrum shortage. Moreover, it can also offload traffic from congested macro base stations (MaBSs) to small base stations (SmBSs). It reduces the number of users competing for communication resources and improves the service quality. Acknowledging these advantages, researchers are studying the multi-tier heterogeneous networks (HetNets) to implement network densification, where different radio access technologies (RATs) working in both licensed and unlicensed spectrums coexist.

### 1.1. Motivation and Related Work

Although network densification can offer greater capacity and offload more data traffic from existing congested networks, the growth of interference among SmBSs accelerates, particularly since the interferers are nearer and larger in number. Interference will then become the biggest constraint to network capacity improvement. Initially, when SmBSs are sparse, increasing the density can provide more available channels and enhance the network capacity. Nevertheless, as the density grows, the interference will heavily degrade the signal-to-interference-plus-noise ratio (SINR) and limits the capacity. It brings an interesting question: is there an optimal SmBS density that maximizes the network capacity?

However, a further issue that needs to be considered is that high SINR (or link capacity) does not necessarily mean high throughput. If the base station is congested, its performance can also be poor. This is why we often lose mobile connection even though it displays full signal strength on our phones. Thus, contrary to intuition, aggressively offloading users to SmBSs is not always right and can lead to severe performance degradation. This is particularly significant for those SmBSs working in unlicensed bands where BSs are operating in listen before talk (LBT) mode and back off if collision happens. In such a situation, load fulfills the same important role as SINR does, for unreasonable load assignment eventually exhausts channel resources and aggravates the throughput. Therefore, how can the offloading be load-aware?

Exploring these two interesting questions is the subject of this work. There is a significant amount of research focusing on the issue of network densification. For example, Samarakoon et al. [6] and Yunas et al. [7] explored the energy efficiency from perspectives of the joint scheduling method and deployment strategies. Chen et al. [8] and Li et al. [9] investigated the backhaul through both theoretical and practical solutions. Other questions in dense network, such as spectrum sharing [10,11], small cell discovery [12], resource management [13,14], mobile caching [15], small cell boundary [16] and the economic issues [17] of densification were also researched in the references. However, these works did not solve the problem of interference, which is one of the biggest problems in dense networks.

Some research has noted the problem of interference. Interference management was considered in [18,19] using cooperative bargaining game and mean-field game, respectively. Soret et al. [20] and Gao et al. [21] proposed time-frequency domain algorithm and power allocation algorithm to coordinate the inter-cell interference. In addition, the same question was considered by Cho et al. [22] using a power control scheme. Nevertheless, these works have not examined the relation between interference and densification, namely how the base station density influences the interference and what the limit of densification is. They are the fundamental questions of interference in ultra-dense networks. To answer the above questions, the calculation of the interference in dense HetNets is necessary. However, it is tricky since the location of base stations in HetNets is random and irregular. A tractable model to describe the location of irregularly deployed BSs is proposed in [23], called Poisson point process (PPP). Based on this model, several key metrics of HetNets performance were studied, such as coverage probability and SINR distribution [24,25,26,27]. Nevertheless, these works have still not given the answer to the optimal base station density for maximizing the capacity of dense network. In addition, all existing research lacks consideration about the load of small cell base stations, especially when these BSs work in contention-based channels. Thus, none can answer the two questions asked above.

### 1.2. Contributions

The contributions of this paper are threefold:

- We have considered the user association and interference problems in Poisson random networks and derived the expression of the expected link rate of the network as a function of SmBS density, which has revealed both the balance between the gain and the interference brought by densification. Based on the analytical results, we find the optimal BS density that maximizes the capacity of the network. Our work indicates that it is not always reasonable to increase the BS density.
- A method is proposed to analyze the throughput of the contention-based channel in random and irregular networks. Unlike the existing literature about analysis of the contention-based channel, which are all based on a given load, the work in this paper deals with the random load caused by the random network. We have found the junction to introduce the Poisson point process into the Markov chain and thus the contention-based load problems can also be solved in random networks. Our analytical results offer the answers on how the network densification can be load-aware in order to avoid performance degradation. Since the unlicensed bands have played an increasingly important role, the work in this paper can apply to many cases.
- Our analytical and simulation results have provided the optimal BS density for deployment of the dense network. After analyzing the effect of the base station power, density and the network load on the performance of network, the optimal deployment density of the base stations are given under different network conditions. The results can provide insights for network densification.

The rest of paper is organized as follows. The system model is detailed in Section 2. Based on this, we analyze and derive the expected link rate and the throughput of networks in Section 3 and Section 4, respectively. In Section 5, the simulations are conducted to verify and describe our analysis and the strategy for network deployment. Section 6 concludes the paper.

## 2. System Model

We consider a two-tier heterogeneous networks consisting of the macro base stations (MaBSs) tier (e.g., cellular network) and the small base stations (SmBSs) tier (e.g., WLAN). MaBSs operate at licensed spectrum while SmBSs work in unlicensed bands. BSs at the same tier share the channel according to the corresponding medium access control (MAC) protocol. We denote the transmit powers of the MaBS (SmBS) as $P_{M}\left(P_{m}\right)$ and the path loss exponent as α. All BSs are open access.

### 2.1. Distribution of BSs in HetNets

**Figure 1:**
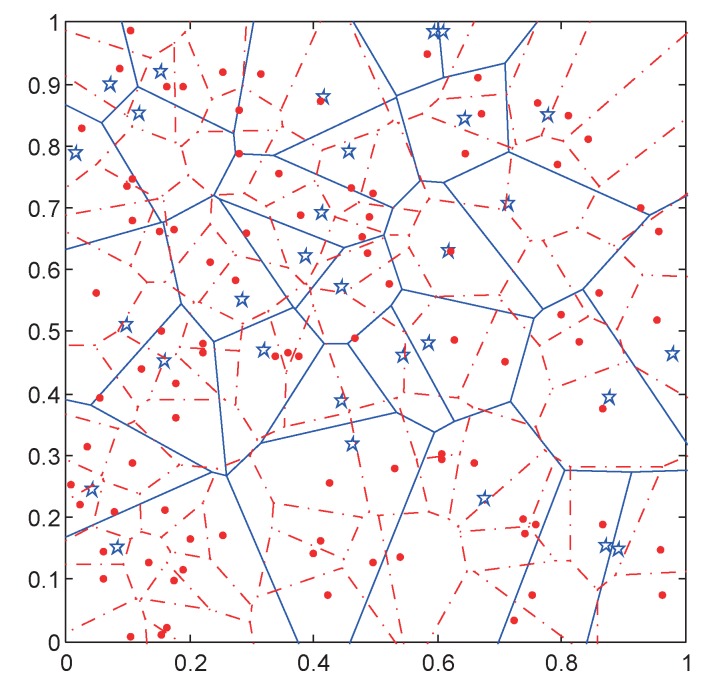
The spatial locations and coverage of macro base stations (MaBSs) and small base stations (SmBSs).

Unlike the traditional network, the BSs in HetNets are deployed irregularly to meet the uneven data demand. Thus, we can use mutually independent PPP to model the spatial locations of the MaBSs, SmBSs and users in HetNets. Its appropriateness has been proved in [23]. PPP offers a method to analyze the performance of HetNets in a statistical point of view. The distribution of MaBSs, SmBSs and users are $\Phi_{M}=\left\{M_{i};\;i=1,2,3,\ldots \right\}$, $\Phi_{m}=\left\{m_{i};\;i=1,2,3,\ldots \right\}$ and $\Phi_{u}=\left\{u_{i};\;i=1,2,3,\ldots \right\},$ respectively, with the corresponding densities $\lambda_{M}$, $\lambda_{m}$ and $\lambda_{u}$. $M_{i}$, $m_{i}$ and $u_{i}$ are the location of the *i*th MaBSs, SmBSs or users. With the PPP model, the two-tier HetNets is abstracted as a Voronoi tessellation with two independent tiers, as shown in Figure 1. The stars stand for the MaBS and their cell ranges are denoted by solid lines. The dots are SmBSs and dashed lines indicate their cell ranges. In such HetNets, each user falls in an overlapping zone of an MaBS and an SmBS, and chooses the BS with strongest long-term received signal strength to connect.

### 2.2. Channel Access Mode of the MaBS Tier

In HetNets, since MaBSs and SmBSs operate at licensed and unlicensed bands, respectively, their channel-sharing modes are also different. For MaBS, we assume the resources (e.g., Long-Term Evolution (LTE) resource blocks) are allocated proportionally among the users. Therefore, a user’s rate is

(1) $$R_{u_{i}}=\frac{W_{M}}{N_{M}}\log\left(1+{\mathrm{SINR}}_{u_{i}}\right).$$

$W_{M}$ is the bandwidth of the MaBS tier and $N_{M}$ is the number of users that are served by a MaBS. Doing summation, we get the throughput of a MaBS as

(2) $$S_{M}=\frac{1}{N_{M}}\sum_{u_{i}=1}^{N_{M}}W_{M}\log\left(1+\mathrm{SIN}R_{u_{i}}\right).$$

It demonstrates that the throughput of a MaBS depends on the SINR distribution of MaBS users. Since both MaBSs and users are distributed in PPP, with the method of Theorem 1 in [23], it can be proved that SINR distribution of the users in MaBS network only depends on the path loss exponent and deployment density of MaBSs. For the practical network, the path loss exponent and density of MaBS are normally determined and fixed so the throughput of the MaBS network does not depend on network densification. On the other hand, since SmBS density is much larger than MaBS density, the areal throughputs of SmBS and MaBS are not in the same order of magnitude. The areal throughput of SmBS is far more than MaBS’s and the areal throughput of MaBS is not notable in overall network performance. Thus, to improve the network throughput, we should focus on improvement of the SmBS network. Actually, in a general case where the base stations of both tiers can be deployed freely, the densities of MaBS and SmBS interact. The variety of MaBS density can directly influence the optimal SmBS density. Since the rate of MaBS is related to the MaBS density, we should calculate the rate of MaBS to derive the total throughput after MaBS density is determined according to the optimal SmBS density. However, when we focus on this paper, some specific assumptions are set in the system model. First, according to the practical network, the MaBSs have been already deployed and the density is fixed. We can only add new SmBSs into the network and can only control SmBS density. Since MaBS density is fixed and due to Theorem 1 of [23], the SINR distribution of MaBS is steady and the average rate of MaBS is also relative steady. It does not impact the rate of MaBS when we optimize the SmBS density. Second, MaBSs and SmBSs operate at different spectrums. It means that these two tiers do not have mutual interference. Thus, the newly added SmBSs do not degrade the MaBS’s’ SINR. With these specific conditions, it is different from the general case. It allows us just to optimize deployment of SmBS in the HetNets.

### 2.3. Channel Access Mode of the SmBS Tier

**Figure 2:**
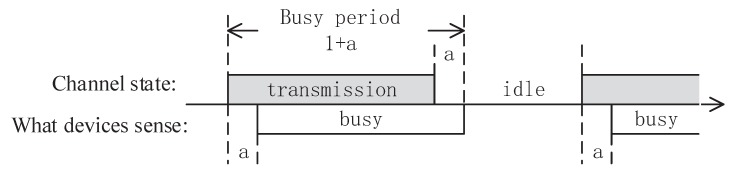
Channel state in a non-persistent carrier sense multiple access (CSMA) network.

SmBSs operate in unlicensed bands where the bandwidth is $W_{m}$. We assume that they work in LBT mode. A slotted non-persistent carrier sense multiple access (CSMA) protocol is adopted in the SmBS tier for channel sharing. Since the fairness of the resource allocation is not the primary question we concern in network deployment and is beyond the scope of this paper, we assume that all user devices in the SmBS tier are the same and the rate is the average ergodic rate in the small cell. All SmBS users have the same priority, contention window size and scheduling tag. Thus, the channel can be allocated fairly by the protocol. For ease of analysis, we include the acknowledgement (ACK) packet in transmission data. *a* is the ratio of channel propagation time to packet transmission time. It depicts the channel state of the SmBS tier in Figure 2. The channel propagation time and transmission time are normalized as *a* and 1.

In the SmBS tier, users firstly sense the channel and then send packets only when the channel is sensed idle. If a collision occurs, the packets will be retransmitted according to K-Exponent back off algorithm. For this algorithm, a backlogged packet transfers to state *i* after its *i*th collision and will be scheduled to retransmit with an exponent probability $q^{i}$, where i=1,2,…K, 0<q<1, and *K* is the end state. The contention-based channel shared among the SmBS and its users can be regarded as a multi-queue single-server traffic model. The data of all devices (SmBS and users) are Geo/G/1 queues with a Poisson arrival process. Thus, the fresh and retransmission packets from all devices form a Poisson stream in the channel. We assume the average arrival rate of a device is $\lambda_{\mathrm{data}}$ and denote the total arrival rate as *G*. Since the SmBSs are far enough from each other given their low transmit power, the received power from devices in other small cells is lower than the channel sensing threshold. Therefore, we do not consider the inter-cell contention.

The channel has three states, namely ‘idle’, ‘successful transmission’ and ‘collision’. We denote the limiting probability of them as $\pi_{\mathrm{idle}}$, $\pi_{\mathrm{suc}}$ and $\pi_{\mathrm{col}},$ respectively. $\pi_{\mathrm{idle}}$ is $e^{-aG},$ indicating that there is no channel access attempt in duration *a*. $\pi_{\mathrm{suc}}$ is $aGe^{-aG}$ since the transmission is successful when there is only one device sending data. Therefore, $\pi_{\mathrm{col}}$ is $1-e^{-aG}-aGe^{-aG}$. The duration of these three state are, respectively, $t_{\mathrm{idle}}=a$, $t_{\mathrm{suc}}=1+a$ and $t_{\mathrm{col}}=1+a$.

Among the three states, the channel is utilized only in the successful transmission state. Otherwise, the channel is either idle or backing off. Thus, the channel is productive only in a portion of time. Let $f_{m}$ be the portion of the time that there is a successful transmission in the channel and the expected link rate of the channel is *R*. Therefore, the throughput of an SmBS is

(3) $$S_{m}=R\cdot f_{m}.$$

To analyze and maximize the throughput of SmBS, we need to solve two problems:

1. the expected link rate *R*;
2. the productive fraction of time $f_{m}$.

By controlling the parameters that influence *R* and $f_{m}$, the maximization of $S_{m}$ can be realized.

## 3. The Expected Link Rate of the SmBS Tier

In this section, we derive the expected ergodic link rate of the SmBS tier and analyze what and how factors of deployment influence the link rate. For the SmBS network that is dense and irregular, there are two important questions in the analysis of link rate. They are the user association and interference distribution in the SmBS tier. User association determines the distance between the user and its serving BS, and the amount of data that can be offloaded to the SmBS tier. Interference distribution plays the key role in SINR.

### 3.1. Distribution of the Distance from a User to Its Serving BS in the SmBS Tier

Without loss of generality, we assume a user is at the origin. In the SmBS tier, since the transmit power in the same tier is identical, the SmBS closest to the user offers the strongest received power so the user will associate with it. $R_{m}$ denotes the distance between them. Its distribution can be obtained from the following lemma.

**Lemma** **1.** *The probability distribution function (PDF) of the distance from the user to its serving SmBS is*

(4) $$f_{R_{m}}\left(r\right)=2\pi \lambda_{m}r\exp\left(-\pi \lambda_{m}r^{2}\right).$$

**Proof**  **of Lemma 1.** The result can be proofed by modifying the proof of Lemma 3 of [24]. ☐

Equation (4) indicates that $R_{m}$ is more likely to be small as the density $\lambda_{m}$ increases. This is because users can find and connect to an SmBS more easily when the SmBS network becomes denser.

### 3.2. Probability of a User Connecting to the SmBS Tier

Since a user chooses a BS to connect depending on the strongest received power, the condition that a user is associated with the SmBS tier is $BP_{m}R_{m}^{-\alpha }>P_{M}R_{M}^{-\alpha }$, where *B* is the bias of the SmBS tier and $R_{M}$ is the distance from the user to the nearest MaBS. Thus, the probability that a user is associated with the SmBS tier can be obtained as follows.

**Lemma** **2.** *A user connected to the SmBS tier with the probability $A_{m}$ which is*

(5) $$A_{m}=\frac{\lambda_{m}}{\hat{C}\lambda_{M}+\lambda_{m}},$$

*where $\hat{C}={\left(\frac{P_{M}}{BP_{m}}\right)}^{\frac{2}{\alpha }}$.*

**Proof** **of Lemma 2.** See Appendix A. ☐

Since the fairness of resource allocation is not the main topic of this paper, we assume B=1 unless explicitly stated. B=1 means that users have no preference to select any certain tier. We will discuss the problem of fairness in Section 4.4, where *B* can take other values.

Equation (5) indicates that SmBS density and the transmit power of SmBS have the influence on network association. Users are more likely associated with the SmBS tier when SmBS network gets denser and its transmit power becomes stronger.

### 3.3. Interference Distribution in the SmBS Tier

In the SmBS tier, recall that the user chooses an SmBS offering the strongest received power to connect, thus all other SmBSs interfere the user. The interference can be described as

(6) $$I=\sum_{i\in \Phi_{m}/b_{0}}P_{m}r_{i}^{-\alpha },$$

where $b_{0}$ is the serving SmBS of the user and $r_{i}$ is the distance from *i*th SmBS to the user.

**Lemma** **3.** *The PDF of the interference (when α=4) in the SmBS tier is*

(7) $$f_{I}\left(I\right)=\frac{e^{-\frac{\pi^{3}P_{m}{\lambda_{m}}^{2}}{4I}}\pi \lambda_{m}P_{m}^{1/2}}{2I^{3/2}}.$$

**Proof** **of Lemma 3.** See Appendix B.  ☐

With the interference distribution, we can further derive the expected value of the link rate.

### 3.4. The Expected Link Rate of the SmBS Tier

With the Equations (4), (5) and (7), we can get the expected link rate of the SmBS tier based on the Shannon formula. The expression of the expected link rate of the SmBS tier is

(8) $$R=A_{m}\int_{\eta }^{\infty }\int_{\eta }^{\infty }f_{I}\left(I\right)\;\;\;W_{m}\log\left(1+\frac{P_{m}r^{-\alpha }}{I+\sigma^{2}}\right)f_{R_{m}}\left(r\right)drdI,$$

where η→0. $\sigma^{2}$ is the power of noise. Considering the propagation environment where the small cells are deployed, according to [28], we assume α=4. Thus, we have

(9) $$R=\pi^{2}W_{m}\frac{\lambda_{m}^{3}P_{m}^{1/2}}{\hat{C}\lambda_{M}+\lambda_{m}}\int_{\eta }^{\infty }\int_{\eta }^{\infty }\frac{e^{-\frac{\pi^{3}P_{m}\lambda_{m}^{2}}{4I}}}{I^{3/2}}r\exp\left(-\pi \lambda_{m}r^{2}\right)\log\left(1+\frac{P_{m}r^{-4}}{I+\sigma^{2}}\right)drdI.$$

It is observed that the link rate is the function of SmBS density. Thus, by selecting the reasonable deployment density, the link rate can be optimized.

## 4. Throughput of MAC Layer in SmBS Tier

Since SmBSs are working in LBT mode, the factors that influence the network performance are not only link rate but also the load of an SmBS. Aggressively offloading data to the SmBS tier is not reasonable since heavy load incurs frequent collision and backoff, and degrades the utilization of the channel. Thus, it is necessary to work out load-aware offloading strategies to make full use of the channel and maximize the throughput.

### 4.1. Fraction of Time for a Productive Channel.

**Figure 3:**
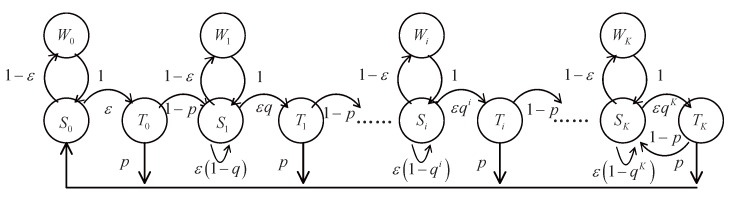
State transition diagram of a packet in the SmBS channel.

Described in the system model, the state transition diagram of a packet in the SmBS channel is depicted in Figure 3 (and more detailed state transition analysis can also be found in [29]). There are three states for each packet. Depending on the collision times *i*, we use $S_{i}$ (sensing), $W_{i}$ (waiting) and $T_{i}$ (transmission) to denote the states, where i=0,1,2,…K. We assume ε to be the probability that the channel is sensed idle and *p* is the successful probability of transmission. Considering a packet in state *i*, it transfers from $S_{i}$ to $W_{i}$ if the channel is sensed busy. We assume the time of $W_{i}$ is equal to packet transmission time. When the channel is detected as idle, the packet will transfer from $S_{i}$ to $T_{i}$ with the probability $q^{i}$. If it is successfully sent, a new packet will begin again at the initial state $S_{0}$. Otherwise, it goes to $S_{i+1}$ and continues to sense the channel. The channel is sensed idle if there is no transmission. Thus, its probability is

(10) $$\varepsilon =\frac{a\pi_{\mathrm{idle}}+a\pi_{\mathrm{suc}}+a\pi_{\mathrm{col}}}{a\pi_{\mathrm{idle}}+\left(1+a\right)\left(\pi_{\mathrm{suc}}+\pi_{\mathrm{col}}\right)}=\frac{a}{1+a-e^{-aG}}.$$

The packet transmission is successful only if all other devices do not send data in the duration of *a* at the beginning of each busy period of the channel. Thus, it has $p=e^{-aG}$.

We denote $s_{i}$, $w_{i}$ and $t_{i}$ as the limiting probability of $S_{i}$, $W_{i}$ and $T_{i}$. With the Markov chain in Figure 3, we can derive the following results:

(11) $$\begin{array}{c} s_{0}=w_{0}+pt_{0}+pt_{1}+\cdots +pt_{K}\\ w_{0}=\left(1-\varepsilon \right)s_{0},t_{0}=\varepsilon s_{0};\\ \mathrm{when}\;\;i=1,2,\ldots ,K-1\\ \left\{\begin{array}{c} s_{i}=\varepsilon \left(1-q^{i}\right)s_{i}+w_{i}+\left(1-p\right)t_{i-1}\\ w_{i}=\left(1-\varepsilon \right)s_{i},t_{i}=\varepsilon q^{i}s_{i}; \end{array}\right.\\ s_{K}=\varepsilon \left(1-q^{K}\right)s_{K}+w_{K}+\left(1-p\right)t_{K-1}+\left(1-p\right)t_{K}\\ w_{K}=\left(1-\varepsilon \right)s_{K},t_{K}=\varepsilon q^{K}s_{K}. \end{array}$$

For network stability, let p+q>1, so all states in Markov Chain are positive recurrent. Combining (11) and the duration of the three states $t_{T_{i}}=1$, $t_{W_{i}}=1$ and $t_{S_{i}}=a$, the time-average probability of the states is expressed by

(12) $$\begin{array}{c} \mathrm{when}\;\;i=0,1,\ldots ,K-1\\ \left\{\begin{array}{c} {\tilde{s}}_{i}=ap\left(p+q-1\right){\left(1-p\right)}^{i}/\left(q^{i}D\right)\\ {\tilde{t}}_{i}=\varepsilon p\left(p+q-1\right){\left(1-p\right)}^{i}/D\\ {\tilde{w}}_{i}=\left(1-\varepsilon \right)p\left(p+q-1\right){\left(1-p\right)}^{i}/\left(q^{i}D\right) \end{array}\right.\\ {\tilde{s}}_{K}=a\left(p+q-1\right){\left(1-p\right)}^{K}/\left(q^{K}D\right)\\ {\tilde{t}}_{K}=\varepsilon \left(p+q-1\right){\left(1-p\right)}^{K}/D\\ {\tilde{w}}_{K}=\left(1-\varepsilon \right)\left(p+q-1\right){\left(1-p\right)}^{K}/\left(q^{K}D\right), \end{array}$$

where $D=\left(1+a-\varepsilon \right)\left[pq-\left(1-q\right){\left(1-p\right)}^{K+1}/q^{K}\right]+\varepsilon \left(p+q-1\right)$.

**Lemma** **4.** *The fraction of time that there is a successful transmission in the channel is*

(13) $$f_{m}=\frac{aGe^{-aG}}{1+a-e^{-aG}}.$$

**Proof** **of Lemma 4.** The result can be proofed using the method of Theorem 1 in [29].  ☐

It is observed that the successful-transmission time fraction $f_{m}$ depends on the channel load *G*. It can be understood intuitively since the channel load (also the load of an SmBS) has a direct impact on channel collision in LBT mode. Excessive load will lead to frequent collisions and backoff, and degrade the utilization of the channel. Thus, we need to analyze the channel load *G*.

### 4.2. Analysis of the Channel Load

To obtain the channel load, we need to analyze the average number of users that connect to an SmBS. Based on the aforementioned analysis of user association, it has the following results.

**Lemma** **5.** *The average load of an SmBS is obtained by*

(14) $$G=\frac{\lambda_{u}\lambda_{\mathrm{data}}}{\hat{C}\lambda_{M}+\lambda_{m}},$$

*where $\hat{C}={\left(\frac{P_{M}}{BP_{m}}\right)}^{\frac{2}{\alpha }}$.*

**Proof** **of Lemma 5.** See Appendix C.  ☐

Lemma 5 gives the insight for channel load control. Besides some objective parameters, the load of the SmBS depends on its density $\lambda_{m}$. As the density of the SmBS increases, there are more SmBSs and their average load decreases.

### 4.3. Throughput Analysis of an SmBS Cell

Combining Equations (13) and (14), we then get the throughput of the SmBS as follows:

(15) $$f_{m}=\frac{a\lambda_{u}\lambda_{\mathrm{data}}e^{-\frac{a\lambda_{u}\lambda_{\mathrm{data}}}{\hat{C}\lambda_{M}+\lambda_{m}}}}{\left(\hat{C}\lambda_{M}+\lambda_{m}\right)\left(1+a-e^{-\frac{a\lambda_{u}\lambda_{\mathrm{data}}}{\hat{C}\lambda_{M}+\lambda_{m}}}\right)}.$$

According to Equation (3), the throughput of an SmBS can be given as

(16) $$S_{m}=\frac{2\pi^{2}W_{m}aP_{m}^{1/2}\lambda_{u}\lambda_{\mathrm{data}}e^{-\frac{a\lambda_{u}\lambda_{\mathrm{data}}}{\hat{C}\lambda_{M}+\lambda_{m}}}\lambda_{m}^{3}}{{\left(\hat{C}\lambda_{M}+\lambda_{m}\right)}^{2}\left(1+a-e^{-\frac{a\lambda_{u}\lambda_{\mathrm{data}}}{\hat{C}\lambda_{M}+\lambda_{m}}}\right)}\kappa \left(\lambda_{m}\right),$$

where $\kappa \left(\lambda_{m}\right)=\int_{\eta }^{\infty }\int_{\eta }^{\infty }\frac{e^{-\frac{\pi^{3}P_{m}\lambda_{m}^{2}}{4I}}}{2I^{3/2}}r\exp\left(-\pi \lambda_{m}r^{2}\right)\log\left(1+\frac{P_{m}r^{-4}}{I+\sigma^{2}}\right)drdI$.

From Equation (16), it is observed that $S_{m}$ is a function of SmBS density $\lambda_{m}$. It means that we can maximize the throughput $S_{m}$ via controlling $\lambda_{m}$. Since it does not have a simple form for $\kappa (\lambda_{m})$, for fast calculation of the optimal SmBS density $\lambda_{m}$, we propose a fitting function to approach $\kappa (\lambda_{m})$, which is shown in Appendix D.

It is worth noting that some objective parameters that indicate the condition of network, such as the power of SmBS $P_{m}$ and the user density $\lambda_{u}$, also have influence on $S_{m}$. Thus, $S_{m}$ should also be analyzed under different network conditions. We will do it in Section 5.

### 4.4. The Fairness of the Tier Association

Fairness of resource allocation is an important problem in practical networks. Because ensuring user-level fairness would involve specific scheduling strategies and make the analysis intractable, we discuss a simple tier-level fairness strategy in this paper. The network in this paper is a two-tier network, so the fairness of the connection to different tiers is also an interesting question. In the HetNets, the rates of MaBS and SmBS tiers may differ and the user may have different user experience when connecting to different tiers. Thus, the fairness problem is to let the users have the same average rate no matter which tier they connect to. This is equivalent to requiring both tiers to have the same effective areal throughput (total throughput divided by effective cell area). A technology called Cell Range Expansion (CRE) [30,31] has been proposed to control the fairness of the association. CRE makes the user association biased by adding an association bias. To be more specific, within the two available tiers for a user to associate, the index of the chosen tier is

(17) $$\mathrm{index}=\arg\max_{M,m}\left(P_{M}R_{M}^{-\alpha },BP_{m}R_{m}^{-\alpha }\right),$$

where *B* is the bias of the SmBS tier. With the association bias, it effectively expands $\left(B>1\right)$ the effective coverage area of SmBSs when they have a larger rate than MaBSs, or decreases $\left(B<1\right)$ the effective coverage area of SmBSs when they have less rate than MaBSs.

For the fairness of the users associated with both tiers, their average rates should be same. Thus, we have

(18) $$\frac{S_{M}Q_{\mathrm{area}}\lambda_{M}}{\left(1-A_{m}\right)Q_{\mathrm{area}}}=\frac{S_{m}Q_{\mathrm{area}}\lambda_{m}}{A_{m}Q_{\mathrm{area}}},$$

where $Q_{\mathrm{area}}$ is the total area of the HetNets and $S_{M}$ is the throughput of MaBS. The left side of the equation can be deduced using the method in Theorem 2 of [24]. Based on Equation (18), we can obtain an equation of *B*. By solving it using search algorithms, we can get the association bias *B*. Since the fairness of allocation is not the main topic of this paper, the detailed research of the fairness and its algorithms will be presented in our future work.

With the bias *B*, the users will have the same rate no matter to which tier they associate. Hence, fairness between the two tiers is guaranteed. However, CRE technology is only a suboptimum solution for network throughput [31]. Deployers of the HetNets need to choose CRE or the optimal solution by deciding what is most suitable with their demand.

## 5. Numerical Results and Discussion

The density of SmBS is a key parameter of the network densification. With the analysis, it is observed that SmBS density not only influences the link rate of the heterogeneous network but also impacts the utilization of the channel. In this section, we will first reveal the effect of SmBS density on the link rate and the channel efficiency of the small cell network via simulation. Then, the relation between SmBS density and the throughput of the SmBS will be described under different network conditions. With these results, the insights for the deployment of the heterogeneous network can be obtained.

In the simulation, we divide the region into meshes of which the area is 300m×300m. We take the mesh as the unit area in the simulation. Since the area of a mesh is just the coverage of the MaBS in metropolis [32], we place one MaBS in each mesh. MaBS configurations are set based on [32]. The transmit power of MaBS is 20 W. We assume omnidirectional antennas and large-scale channel fading with path loss exponent value of 4 are invoked in both tiers of HetNets [28]. We set the bandwidth of the SmBS tier as 10 MHz and the additive noise is $10^{-6}$ W (the power density is −100 dBm/Hz). The results are obtained as follows.

### 5.1. Effect of SmBS Density on the Link Rate

**Figure 4:**
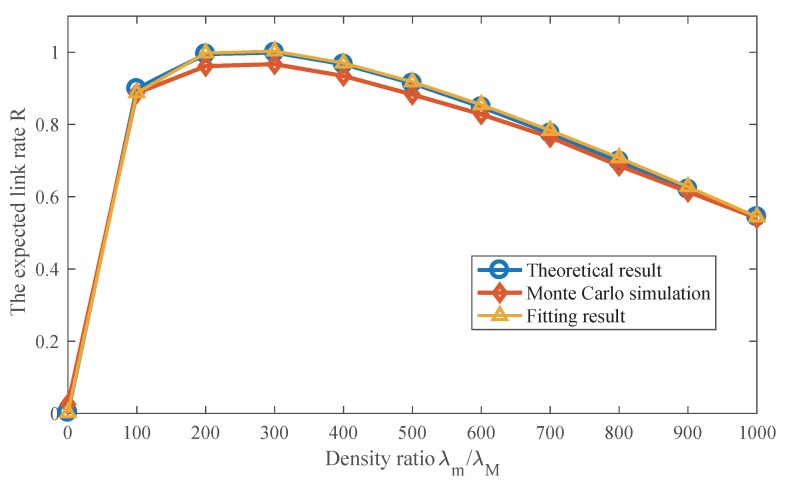
Comparison between the theoretical result and Monte Carlo simulation. $P_{m}/P_{M}\;=\;0.001$.

We conduct the Monte Carlo simulation to verify the analytical results and draw the simulation and theoretical results. The theoretical results are generated using Equation (9). As Figure 4 shows, the results match well. The figure shows the effect of $\lambda_{m}$ on the expected link rate of the SmBS tier. As SmBS density increases, there are three phases for the link rate. In the first phase, the link rate increases with SmBS density, but the speed of growth gradually decreases. In this phase, when there are denser SmBSs, more data traffic can be offloaded to the SmBS tier. Meanwhile, denser SmBSs largely shorten the distance from a user to its associated SmBS, which offers better signal quality. Though it can also incur more interference, the gain outperforms the cost in this phase. However, since the interference accelerates, the increase speed of the link rate slows down. The second phase is the peak of the link rate, where the gain equals the interference cost. The horizontal axis of the peak point is the optimal SmBS density for the link rate. Then, in the third phase, the interference accelerates at a greater rate as SmBSs become denser and overwhelms the gain that densification brings. It indicates that superfluous BSs can degrade the network performance instead of gain. The curve fitting result is also provided in Figure 4, and it indicates that the fitting results matches the theoretical results well.

**Figure 5:**
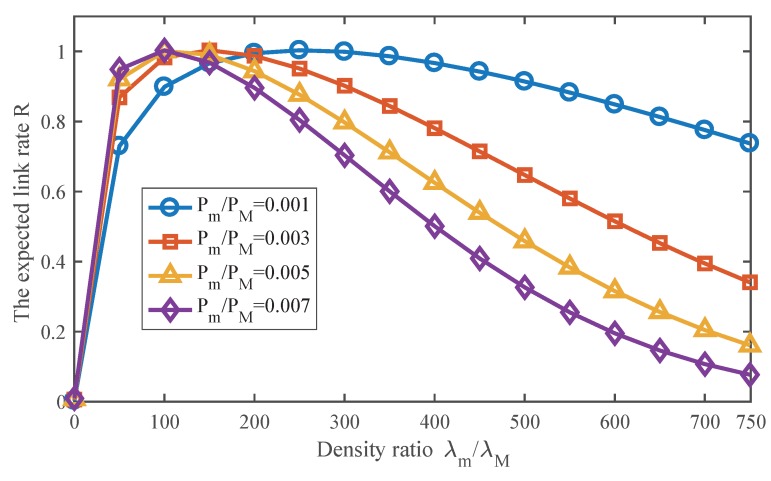
The effect of SmBS density on the link rate under different SmBS transmit powers.

With Equation (9), Figure 5 shows the effect of SmBS density on the link rate under different SmBS transmit powers. There is always an optimal SmBS density when SmBS transmit power varies. However, the transmit power indeed has a significant influence on SmBS deployment. As SmBS transmit power increases, the link rate increases and decreases faster, and the optimal density also decreases. The reason why the link rate increases faster is that SmBS with stronger transmit power offers better signal quality. However, when SmBS is of high density, stronger transmit power also accelerates interference at a faster rate, which degrades the link rate sharply. It is also observed that the SmBS with lower transmit power has higher optimal deployment density. It is consistent with intuition that we can deploy more BSs if we reduce the transmit power. Although denser SmBSs cannot enhance the maximum rate of each link, they can offer more SmBSs to suffer the network load, which contributes to the network performance. We will talk about the load in the next section.

### 5.2. Effect of SmBS Density on the Channel Efficiency

**Figure 6:**
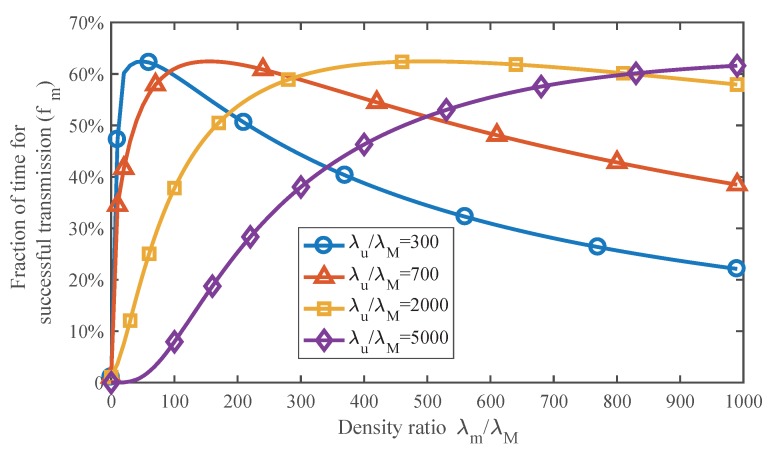
The effect of SmBS density on the channel efficiency under different user densities. $P_{m}/P_{M}\;=\;0.001$.

Figure 6 indicates how the SmBS density influences the channel efficiency, which is described by the fraction of time for successful transmission. The user density demonstrates the load of the network, for example busy or idle. The results are generated by (15). For different user densities, there are optimal SmBS densities maximizing the channel efficiency. When SmBSs are sparse, the average load of them will be high, which can lead to frequent collision in the channel and degrading the performance. As we deploy more SmBSs, the performance will improve. However, if there are too many SmBSs, the performance will decrease again due to the low utilization of the channel. It can also be observed that the optimal SmBS density increases with the user density. This is because more SmBSs are needed to suffer the heavy load of network. However, note that always increasing the SmBS density in busy networks is not correct, since excessive SmBSs result in huge interference. We will consider the balance later in this paper.

### 5.3. The Areal Throughput of HetNets and the Optimal Deployment Scheme

As aforementioned, it is not only the link rate but also the channel efficiency that has impact on the throughput of SmBS. Unlike existing literature, we analyze and simulate the impact of both of them, which gives many insights for densification deployment. With the parameter settings mentioned at the beginning of this section, we simulate a two-tier HetNet consisting of MaBSs and SmBSs. The term of total HetNets throughput (the sum of the throughput of all MaBSs and SmBSs) and the term of total HetNet areal throughput that is obtained by dividing the total HetNets throughput by the total HetNets area are selected to indicate the performance of the network.

**Figure 7:**
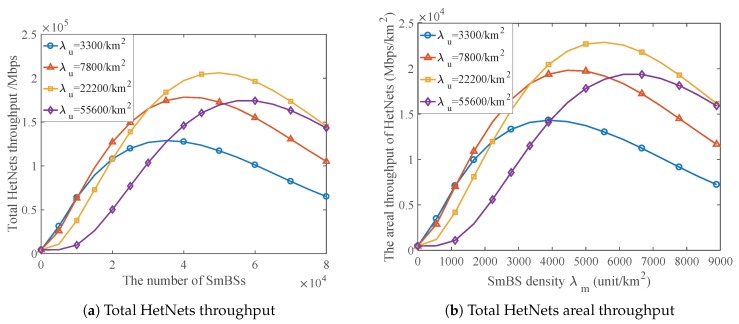
The impact of SmBS density on HetNets throughput. Network area = $9\;km^{2}$, $P_{m}=60\;\mathrm{mW}$.

Figure 7a shows the total HetNets throughput. The network area in the simulation is $9\;km^{2}$, where 100 MaBSs are deployed in this area. We have simulated the performance of HetNets under different user densities and SmBS densities. The simulation results show the sum throughput of all MaBSs and SmBSs in this simulation area. With these results and dividing the total HetNets throughput by the total HetNets area, we can obtain the total HetNets areal throughput.

Figure 7b illustrates the impact of SmBS density on the total HetNets areal throughput. As the figure shows, deploying more SmBSs can offer greater total areal throughput since it provides more access and reduces resource competition. However, always increasing SmBS density is not correct due to interstation interference. We can find that the optimal deployment density of SmBS always exists under different network load conditions that are described by the user density. In addition, as the user density varies, we can observe that higher user density needs higher SmBS density, which is consistent with the intuition.

**Figure 8:**
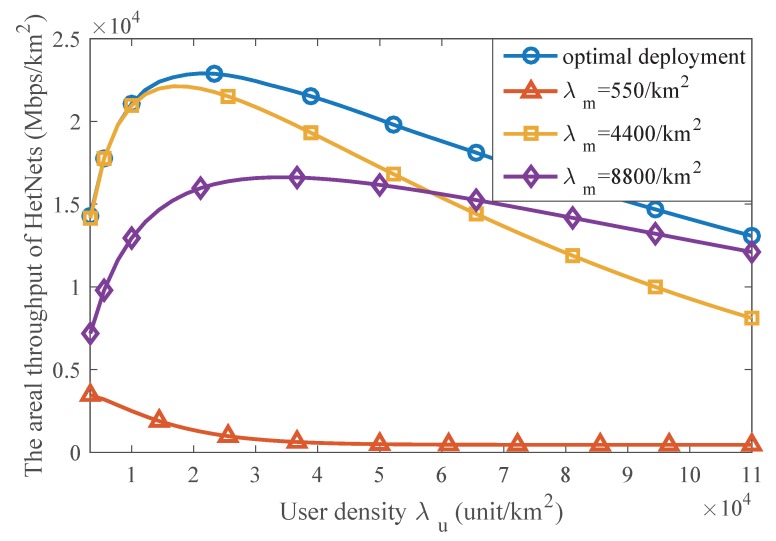
Comparison between the optimal densification deployment and suboptimal ones. $P_{m}=60\;\mathrm{mW}$.

Figure 8 has compared the performance of the optimal deployment and other suboptimal ones. The optimal deployment is implemented by selecting the optimal SmBS density under different network conditions of user density. We take $\lambda_{m}=550/km^{2}$, $\lambda_{m}=4400/km^{2}$ and $\lambda_{m}=8800/km^{2}$ as examples of the suboptimal deployment, which indicate low density, medium density and relative high density. When the SmBSs are of low density, HetNets performance deteriorates rapidly as the user density increases because such few SmBSs cannot afford too many users. When the SmBSs are of higher density, they perform better than those of lower density do in the crowded network but act more poorly in the light-loaded network, since denser SmBSs have greater capability to offer service to massive users, and, meanwhile, they can also incur heavy interference. With the comparison, we find that the optimal deployment scheme outperforms other ones and achieves the best performance under any user density.

### 5.4. The Throughput of an SmBS and the Optimal Deployment Scheme

Although the areal throughput of HetNets can indicate the performance of the networks, there is another metric for optimization, which is the throughput of each SmBS. This metric focuses on the efficiency of the SmBS and aims to deploy the base stations in a cost-effective way. This metric also attracts attention of some telecom operators. Thus, we also investigate the throughput of an SmBS and find the optimal density ratio of SmBSs and MaBSs.

**Figure 9:**
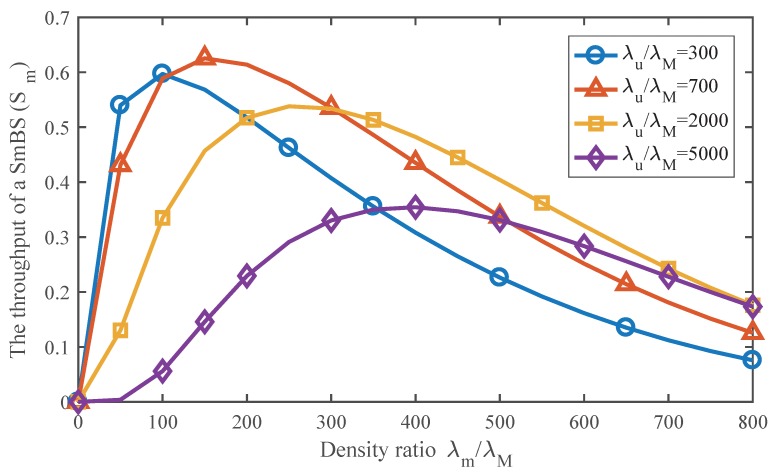
The impact of SmBS density on the throughput of an SmBS. $P_{m}/P_{M}=0.003$.

With Equation (16), Figure 9 illustrates the impact of SmBS density on the throughput of an SmBS. From the picture, there is always an optimal SmBS density under different user densities. As the user density increases, more SmBSs are needed to avoid overload. In the network that is not too busy, i.e., $\lambda_{u}/\lambda_{M}=300$ or $\lambda_{u}/\lambda_{M}=700$, denser SmBSs can offload more data traffic, so the maximum throughput increases with the user density. However, in busy network, i.e., $\lambda_{u}/\lambda_{M}=2000$ or $\lambda_{u}/\lambda_{M}=5000$, since the optimal SmBSs densities are already very dense, higher SmBS density can lead to greater interference that degrades the link rate and further reduces the throughput of an SmBS, so the maximum throughput of an SmBS decreases with the user density in such situation. Nevertheless, as depicted in Figure 9, when $\lambda_{u}/\lambda_{M}\;=\;700$, the optimal SmBS density is $150\lambda_{M}$, and if it increases to $\lambda_{u}/\lambda_{M}\;=\;5000$, the SmBS density should also be enhanced to $400\lambda_{M}$ in order to achieve the maximum throughput of an SmBS. It indicates that network densification is reasonable.

**Figure 10:**
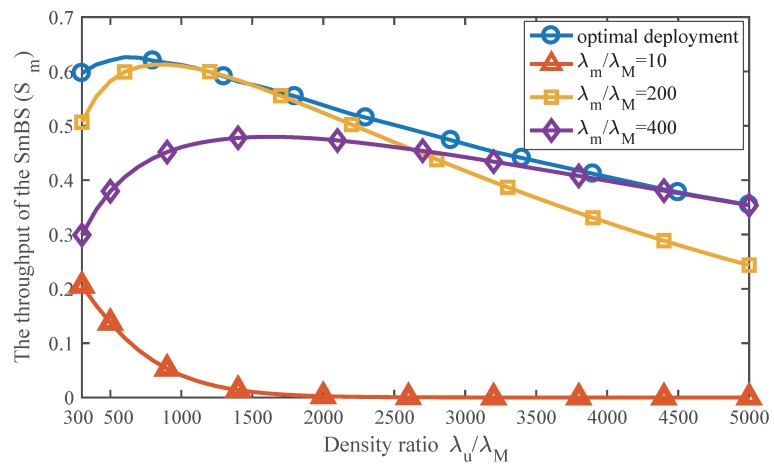
Effect of the optimal deployment on the throughput of an SmBS. $P_{m}/P_{M}=0.003$.

Figure 10 has compared the performance of the optimal deployment and other suboptimal ones. The optimal deployment is implemented by selecting the optimal SmBS density under different network conditions of user density. We take $\lambda_{m}/\lambda_{M}=10$, $\lambda_{m}/\lambda_{M}=200$ and $\lambda_{m}/\lambda_{M}=400$ as examples of the suboptimal deployment, which indicate low density, medium density and relative high density. As the figure shows, our deployment scheme outperforms other ones and achieves the best performance of an SmBS under any user density.

## 6. Conclusions

In this paper, we present a tractable method to analyze the heterogeneous network (HetNets) densification and provide the optimal deployment strategy that maximizes the network throughput. The expected link rate is deduced by considering both association and interference problems in heterogeneous networks. Then, we solve the contention-based load problem in a random network by introducing the Poisson point process to the Markov chain. Based on this, by considering both physical and MAC layers, the throughput of the small base stations is derived. The analytical result indicates the relation among the network performance, base station density, transmit power and user density; meanwhile, it offers a method to calculate the optimal base station density that maximizes the performance. Simulations are conducted to verify and illustrate the analysis. Our work can provide insights for network densification and guidance for network deployment.

## Appendix A

**Proof**  **of Lemma 2.** A user connects to the SmBS tier when $BP_{m}R_{m}^{-\alpha }>P_{M}R_{M}^{-\alpha }$, where *B* is the bias of the SmBS tier. Thus, we have

(A1) $$A_{m}=P\left\{BP_{m}R_{m}^{-\alpha }>P_{M}R_{M}^{-\alpha }\right\}=\int_{r>0}\underset{A}{\underset{︸}{P\left\{R_{M}>{\left(\frac{P_{M}}{BP_{m}}\right)}^{\frac{1}{\alpha }}r\right\}}}f_{R_{m}}\left(r\right)dr.$$

Since $R_{M}$ is a random variable and follows the Poisson point process, Equation (A1) can be derived as

(A2) $$A_{m}=\int_{r>0}\exp\left(-\pi \lambda_{M}{\left(\frac{P_{M}}{BP_{m}}\right)}^{\frac{2}{\alpha }}r^{2}\right)f_{R_{m}}\left(r\right)dr.$$

Then, combining Equation (4) with (A2), we have

(A3) $$A_{m}=2\pi \lambda_{m}\int_{r>0}r\exp\left(-\pi \lambda_{M}{\left(\frac{P_{M}}{BP_{m}}\right)}^{\frac{2}{\alpha }}r^{2}\right)\exp\left(-\pi \lambda_{m}r^{2}\right)dr=\frac{\lambda_{m}}{\hat{C}\lambda_{M}+\lambda_{m}},$$

where $\hat{C}={\left(\frac{P_{M}}{BP_{m}}\right)}^{\frac{2}{\alpha }}$. This is the result in Equation (5).  ☐

## Appendix B

**Proof** **of Lemma 3.** The typical user at the origin *O* receives the interference from other SmBSs in a circle. Defining the interference in this circle with radius *z* as *I*, we have

(A4) $$I=P_{m}\sum_{m_{i}\in \Phi_{m}\cap b\left(O,z\right)}\ell (∥r∥),$$

where $b\left(O,z\right)$ is the circle and $\ell \left(r\right)=r^{-\alpha }$ is the pass loss function. The lemma can be deduced using the method in [33]. We define the characteristic function of *I* as

(A5) $$F_{I}\left(\omega \right)\overset{\Delta }{\;=}E\left(e^{j\omega I}\right).$$

Given that there are *k* points in b(O,z), these points are distributed on the disk with radial density, which is given by [33]

(A6) $$f_{R}\left(r\right)=\left\{\begin{array}{cc} \frac{2r}{z^{2}}, & 0\leq r\leq z,\\ 0, & \mathrm{otherwise}. \end{array}\right.$$

When there are exactly *k* SmBSs in b(O,z), then

(A7) $$E\left(e^{j\omega I}|\Phi_{m}\left(b(O,z)\right)=k\right)={\left(\int_{0}^{z}\frac{2r}{z^{2}}\exp\left(j\omega P_{m}\ell \left(r\right)\right)dr\right)}^{k}.$$

According to the properties of PPP, the unconditional characteristic function of the interference can be derived as

(A8) $$F_{I}\left(\omega \right)=\sum_{k=0}^{\infty }\frac{\exp\left(-\lambda_{m}\pi z^{2}\right){\left(\lambda_{m}\pi z^{2}\right)}^{k}}{k!}\times {\left(\int_{0}^{z}\frac{2r}{z^{2}}\exp\left(j\omega P_{m}\ell \left(r\right)\right)dr\right)}^{k}.$$

With the Taylor expansion of the sum, it has

(A9) $$F_{I}\left(\omega \right)=\exp\left(\lambda_{m}\pi z^{2}\left(-1+\int_{0}^{z}\frac{2r}{z^{2}}\exp\left(j\omega P_{m}\ell \left(r\right)\right)dr\right)\right).$$

Assuming $\ell^{-1}\left(x\right)$ as the inverse function of ℓ(r), and substituting $r\to \ell^{-1}\left(x\right)$, z→∞, we enlarge the area to infinity:

(A10) $$\lim_{z\to \infty }z^{2}\left(-1+\int_{0}^{z}\frac{2r}{z^{2}}\exp\left(j\omega P_{m}\ell \left(r\right)\right)dr\right)=\int_{0}^{\infty }{\left(\ell^{-1}\left(x\right)\right)}^{2}j\omega P_{m}\exp\left(j\omega P_{m}x\right)dx.$$

Combining Equations (A9) and (A10), it has

(A11) $$F_{I}\left(\omega \right)=\exp\left(j\pi \lambda_{m}\omega P_{m}\int_{0}^{\infty }{\left(\ell^{-1}\left(x\right)\right)}^{2}e^{j\omega P_{m}x}dx\right).$$

Substituting $\ell \left(r\right)=x=r^{-\alpha }$ to Equation (A11), then we have

(A12) $$F_{I}\left(\omega \right)=\exp\left(j\lambda_{m}\pi \omega P_{m}\int_{0}^{\infty }x^{-\frac{2}{\alpha }}e^{j\omega P_{m}x}dx\right).$$

Considering the complex environment of propagation, we assume α=4 [28]. Due to $I=P_{m}x$, we get

(A13) $$f_{I}\left(I\right)=\frac{e^{-\frac{\pi^{3}P_{m}{\lambda_{m}}^{2}}{4I}}\pi \lambda_{m}P_{m}^{1/2}}{2I^{3/2}}.$$

This result is consistent with the result in [34].  ☐

## Appendix C

**Proof** **of Lemma 5.** Without loss of generality, we consider a unit area of the network. Based on the expression of $A_{m}$ in Lemma 2, the mean load of an SmBS is

(A14) $$G=\frac{\lambda_{u}\lambda_{\mathrm{data}}A_{m}}{\lambda_{m}}=\frac{\lambda_{u}\lambda_{\mathrm{data}}}{\hat{C}\lambda_{M}+\lambda_{m}},$$

which gives the result in Lemma 5.  ☐

## Appendix D

Since $\kappa (\lambda_{m})$ is the infinite double integral of a transcendental function, it does not have a simple form for fast calculation. We perform curve fitting to approach $\kappa (\lambda_{m})$ and thus get the empirical formula. Observing the data of $\kappa (\lambda_{m})$, we choose the fitting function as

(A15) $$\kappa_{\mathrm{fit}}=C_{1}\lambda_{m}^{C_{2}}-C_{3}\lambda_{m}^{C_{4}},$$

where $C_{1},\;C_{2},\;C_{3}\;\mathrm{and}\;C_{4}$ are the functions of $P_{m}$. Then, we conduct polynomial fitting to get the functions of $C_{1},\;C_{2},\;C_{3}\;\mathrm{and}\;C_{4}$. Since the maximum power of SmBS (e.g., WLAN) is limited by local regulations, the power range of SmBS that we consider in curve fitting is $P_{m}\in \left[0.001P_{M},0.006P_{M}\right]$. The order of polynomial is carefully selected to avoid both underfitting and overfitting. The results are achieved via MATLAB (R2016b, MathWorks Inc., Natick, MA, USA) and given as follows:

(A16) $$\begin{array}{cc} C_{1}= & -8.5658232\times 10^{14}P_{m}^{6}+1.7940284\times 10^{13}P_{m}^{5}-1.4773754\times 10^{11}P_{m}^{4}+6.2145778\times 10^{8}P_{m}^{3}\\ & -1.3725986\times 10^{6}P_{m}^{2}+1.2067786\times 10^{3}P_{m}+1.1487479, \end{array}$$

(A17) $$C_{2}=-5.9245046\times 10^{6}P_{m}^{3}+7.9721220\times 10^{4}P_{m}^{2}-2.6894727\times 10^{2}P_{m}-1.7117077,$$

(A18) $$\begin{array}{cc} C_{3}=\; & 5.1151868\times 10^{14}P_{m}^{6}-9.2334191\times 10^{12}P_{m}^{5}+6.0667929\times 10^{10}P_{m}^{4}-1.8492164\times 10^{8}P_{m}^{3}\\ & +2.6211819\times 10^{5}P_{m}^{2}-1.3845104\times 10^{2}P_{m}-0.003196, \end{array}$$

(A19) $$\begin{array}{cc} C_{4}= & -2.6498472\times 10^{15}P_{m}^{6}+6.4364873\times 10^{13}P_{m}^{5}-6.1989791\times 10^{11}P_{m}^{4}+3.0240235\times 10^{9}P_{m}^{3}\\ & -7.8793336\times 10^{6}P_{m}^{2}+1.0192004\times 10^{4}P_{m}-5.7462256. \end{array}$$

The goodness of fit is 92.42–99.78% in the range of $P_{m}$.
